# Phosphatidylglyerol Lipid Binding at the Active Site of an Intramembrane Protease

**DOI:** 10.1007/s00232-020-00152-z

**Published:** 2020-11-18

**Authors:** Ana-Nicoleta Bondar

**Affiliations:** Freie Universität Berlin, Department of Physics, Theoretical Molecular Biophysics, Arnimallee 14, 14195 Berlin, Germany

**Keywords:** Lipid–protein coupling, Intramembrane protease, GlpG, Phosphatidylglycerol, Hydrogen bonding

## Abstract

Transmembrane substrate cleavage by the small *Escherichia coli* rhomboid protease GlpG informs on mechanisms by which lipid interactions shape reaction coordinates of membrane-embedded enzymes. Here, I review and discuss new work on the molecular picture of protein–lipid interactions that might govern the formation of the substrate–enzyme complex in fluid lipid membranes. Negatively charged PG-type lipids are of particular interest, because they are a major component of bacterial membranes. Atomistic computer simulations indicate POPG and DOPG lipids bridge remote parts of GlpG and might pre-occupy the substrate-docking site. Inhibition of catalytic activity by PG lipids could arise from ligand-like lipid binding at the active site, which could delay or prevent substrate docking. Dynamic protein–lipid H-bond networks, water access to the active site, and fluctuations in the orientation of GlpG suggest that GlpG has lipid-coupled dynamics that could shape the energy landscape of transmembrane substrate docking.

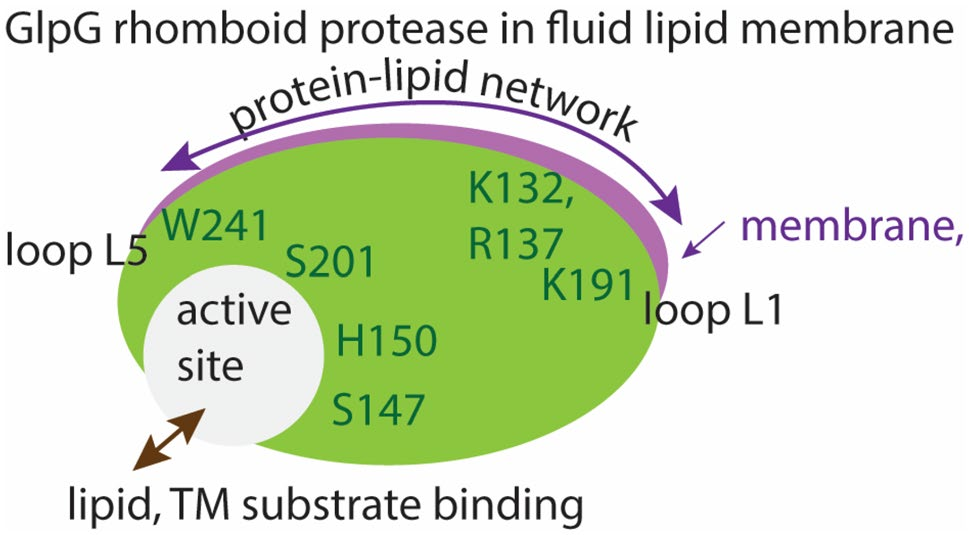

## Introduction

**Fig. 1:**
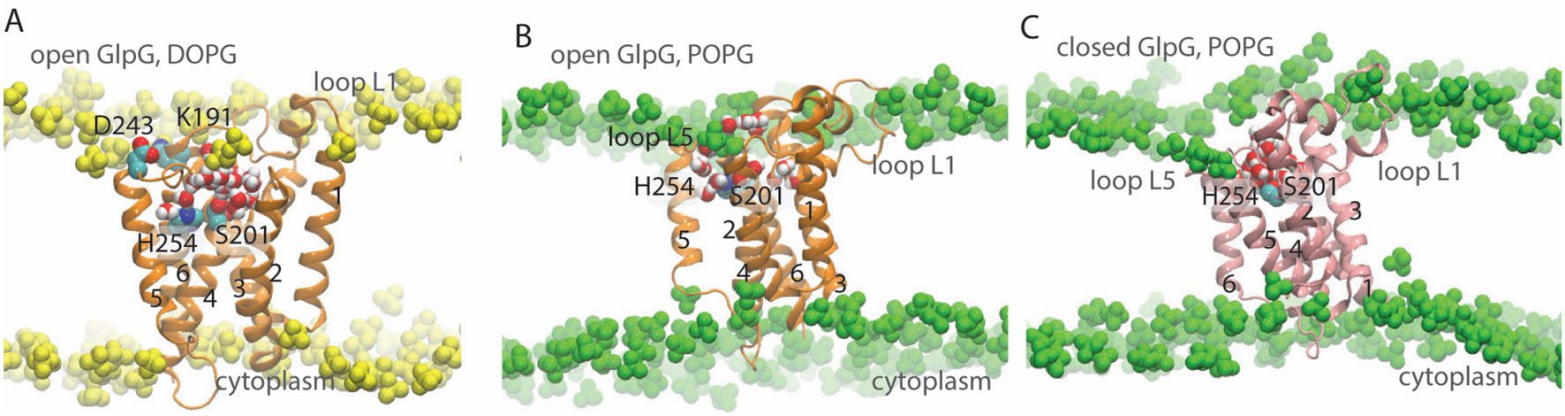
GlpG is a membrane-embedded enzyme whose active site is exposed to lipids and water. Lipid phosphate groups (cut-away view) and water molecules within 9 Å of S201-O*γ* are shown as van der Waals spheres. **a**, **b** Open conformation GlpG in a hydrated DOPG bilayer (panel **a**) and in POPG (panel **b**). **c** Closed conformation GlpG in POPG. All molecular graphics were prepared with VMD (Humphrey et al. 1996)

Rhomboid proteases are membrane-embedded enzymes that cleave substrates to activate signaling paths. These proteases were initially identified in *Drosophila* (Bier et al. 1989), where Rhomboid-1 cleaves the membrane-anchored substrate Spitz, activating the epidermal growth factor (EGF) receptor (Urban et al. 2001). Rhomboid proteases are implicated in, e.g., wound healing (Cheng et al. 2011), cancer (Adrain et al. 2011; Yan et al. 2008; Zou et al. 2009), diabetes (Walder et al. 2005), and malaria infection (Baker et al. 2006). An intriguing feature of rhomboid proteases is that they couple tightly to the membrane: The membrane constrains the folding pathway of the *E. coli* rhomboid protease, GlpG (Schafer et al. 2016), and the catalytic activity of rhomboids depends drastically on the surrounding lipid environment (Urban and Wolfe 2005). Significant impact of the lipid membrane composition on substrate-cleaving properties was also observed for two other membrane-embedded proteases, presenilin, whose transmembrane substrates include amyloid precursor protein (Osenkowski et al. 2008), and signal peptide peptidase, which cleaves signal peptides (Narayanan et al. 2007). The rhomboid protease of *E. coli*, GlpG (Fig. 1), is well characterized by structural biology and biochemistry (for reviews see, e.g., Bondar 2016; Bondar and Lemieux 2019; Brooks and Lemieux 2013; Düsterhöft et al. 2017; Strisovsky 2016; Urban 2010, 2013)) and thus a valuable model system to decipher how lipids shape reaction coordinates of intramembrane proteases. Here, I review and discuss new work on the lipid interactions of GlpG.

A molecular picture of the role of lipids in membrane protein function entails description of how the lipid membrane adjusts to the presence of the protein, of the protein motions in the membrane, and of the structural changes and associated free energy profile along the reaction coordinate of the protein. Deriving such a complete picture would require classical mechanical molecular dynamics simulations to sample the motions of the protein, lipids, and waters in a fluid system at room temperature, and quantum mechanics for reaction coordinates that involve changes in electronic structure, such as breaking and forming of covalent bonds during proteolytic cleavage of a substrate. The classical mechanical simulation trajectory, which gives the time evolution of the Cartesian coordinates of each atom in the simulation system, can be subjected to various analyses to probe conformational dynamics of the protein, to identify specific interactions with potential role in shaping the conformational dynamics of the protein, and to predict and probe mutations that can inform on how the protein works. Activation energy barriers and the reaction energies obtained from quantum mechanical computations can be validated using information about the kinetics of the reaction studied.

Simulations are typically performed on one protein embedded in a bilayer composed of one, or a small number of lipid types and cholesterol, as this enables us to dissect how specific lipid–protein interactions might impact function. By contrast, cell membranes can have a relatively high protein-to-lipid ratio (Marinko et al. 2019) and have complex lipid composition even in bacteria (Op den Kamp et al. 1969; Raetz 1978; Sohlenkamp and Geiger 2016; van Meer et al. 2008) and presence of membrane proteins modulates membrane thickness (Mitra et al. 2004). Recent developments in methodology and computational power have started to enable simulations with complex lipid mixtures (Enkavi et al. 2019; Marrink et al. 2019), which brings about the need of experimental validation of, for example, structural and dynamical properties of various lipid mixtures studied with simulations. Computations on proton-transfer inside a membrane protein would suggest description of the complex lipid membrane composition might be unnecessary, as the energetics of the chemical reaction was largely the same with and without membrane (Adam and Bondar 2018).

Knowledge of the three-dimensional structure of a membrane protein is an essential step towards deciphering its lipid interactions. GlpG is well characterized by X-ray crystallography (White 2006). Briefly, crystallographic structures of GlpG indicate the cap loop L5 and transmembrane (TM) helix 5 are mobile (White 2006), and two conformations denoted as the open and closed conformations are largely distinguished by the orientation TM5, which is displaced laterally in the open conformation (Ben-Shem et al. 2007) (Fig. 1). A conformation of GlpG with an even more pronounced lateral displacement of TM5 was solved in ref. (Wu et al. 2006); here, as in previous work (Bondar 2019), I refer to this structure as open TM5 GlpG.

The first atomistic simulations of open GlpG (Bondar et al. 2009) considered two different lipid membrane environments—1-palmytoyl-2-oleoyl-*sn*-glycero-3-phosphatidylethanolamine (POPE), chosen as model of PE lipids, which in experiments allow GlpG to cleave efficiently the *Drosophila* Spitz substrate (Urban and Wolfe 2005), and 1-palmytoyl-2-oleoyl-*sn*-glycero-3-phosphatidylcholine (POPC) as model of PC lipids, which hinder proteolytic cleavage (Urban and Wolfe 2005).

POPC and POPE have different H-bonding capabilities: whereas the ethanolamine moiety can readily engage in direct H-bonds, the three methyl groups covalently bound to the nitrogen atom of the phosphatidylcholine moiety prevent direct H-bonding to protein. As a consequence, the long L1 loop (Fig. 1b), thought to have an important structural role (Baker et al. 2007), has stronger H-bonds with POPE than with POPC lipids (Bondar et al. 2009). To adjust to the irregular shape and small hydrophobic thickness of GlpG, the membrane thins by up to ~ 3–4 Å, i.e., by about one helical turn (Bondar et al. 2009). Such thinning of about one helical turn is important, as a transmembrane (TM) helical substrate could tilt or unwind in a thin membrane (Bondar et al. 2009; Bondar and White 2012; Wang et al. 2007). Thinning of one-component POPE lipid bilayers in the vicinity of GlpG was confirmed by later simulations of GlpG (Reddy and Rainley 2012; Zhou et al. 2012). Values of the lipid membrane thickness different from the bulk membrane have also been observed, e.g., in simulations of the *E. coli* outer membrane protein FhuA in a 3:1 POPE:POPG lipid bilayer (Goose and Sansom 2013).

Distortion of the lipid membrane surrounding GlpG (Bondar et al. 2009) could explain the unusually rapid diffusion measured for rhomboid proteases in living cells (Kreutzberger et al. 2019). But it remains unclear whether and how a hydrophobic mismatch between GlpG and the membrane indeed impacts significantly how the protein sits in the membrane, its motions, and interactions at the active site.

The thickness of the bulk lipid membrane has a linear dependence on the length of the alkyl chains (Lewis and Engelman 1983), and it will be largely determined by the lipid composition when the concentration of membrane proteins is low (Marinko et al. 2019): Liposomes composed of *E. coli* lipids have a thickness of just 33.5 ± 0.4 Å, whereas the thickness of the *E. coli* cytoplasmic membrane, which contains membrane proteins, is 37.5 ± 0.5 Å (Mitra et al. 2004). Both thickness values measured for *E. coli* (Mitra et al. 2004) are significantly smaller than the ~ 42–45 Å value found for POPE membranes in simulations (Ng et al. 2014). Likewise, the thickness computed for the 3:1 POPE:POPG membrane used as a model of the *E. coli* membrane (Murzyn et al. 2005) is ~ 40-41 Å (Bondar 2019); close to GlpG, POPE:POPG membranes thin by ~ 1–3.8 Å (Bondar 2019). The precise values of the bilayer thickness obtained from simulations with a particular membrane composition may further depend on how the simulations where performed, particularly on the force field used to describe interactions between atoms, and temperature.

The thickness of the *E. coli* membrane (Mitra et al. 2004) is very close to the 37.6 Å and, respectively, 36.6 Å values for the POPG and 1,2-dioleoyl-sn-glycero-3-phosphatidylglycerol (DOPG) overall bilayer thickness (Pan et al. 2014), defined based on differences in the scattering of lipid vs. deuterated water (Kučerka et al. 2011; Pan et al. 2014). PG lipids are incompatible with cleavage of Spitz substrate by GlpG, but not by the homologous YqgP rhomboid protease from *Bacillus subtilis* (Urban and Wolfe 2005), which suggests details in ligand-like interactions between PG and GlpG might contribute to how the catalytic activity of a specific bacterial protease responds to changes in the lipid membrane environment.

That lipids can bind to GlpG as a ligand was suggested by the crystal structure of open GlpG (Ben-Shem et al. 2007), in which a PG lipid headgroup is within H-bond distance from the catalytic groups. Atomistic simulations on open GlpG, initiated without a lipid headgroup at the active site, indicated spontaneous, transient active-site visits by lipid headgroups when GlpG was embedded in POPE or DMPC, whereas lipid headgroups remained outside the active-site region in POPE:POPG (Bondar 2019); instead, POPE lipids bound at the cap loop L5 (Bondar 2019). At the periplasmic side of TM5, H-bonding between D243 (Fig. 1a) and POPE lipids could prevent lipids from approaching closer the active site of GlpG (Bondar 2019).

To dissect interactions between phosphatidylglycerol lipids and GlpG, here I studied the motions of open conformation GlpG in POPG vs. DOPG membranes and of closed conformation GlpG in POPG. The simulations indicate both POPG and DOPG H-bond persistently at or near the active site of open GlpG, where they could compete with substrate docking. By contrast, POPG lipids remain outside of the active-site region of closed GlpG. The internal protein network of GlpG connects to lipids via dynamic H-bonding at both sides of the membrane.

## Methods

*Protein structures for closed and open conformations of GlpG*. For the starting protein coordinates of the closed and open conformation, GlpG I used, respectively, chains A and B from PDB ID:2IRV (Ben-Shem et al. 2007). All titratable amino acid residues were considered in standard protonation states, with Asp and Glu, negatively charged, Arg and Lys, positively charged, and His groups single protonated; H254 was protonated on *Nδ*, and all other His groups were protonated on *Nε*. The crystal structure indicates coordinates for a PG-type lipid headgroup bound at the active site (Ben-Shem et al. 2007). To sample transient binding of bulk lipid headgroups at the active site during simulations, the lipid headgroup was removed from the starting protein structure. 56 and 25 crystal structure waters were included in computations on open and closed GlpG, respectively.

The protein was oriented along the membrane using the Orientations of Proteins in Membranes webserver (Lomize et al. 2011) and placed in hydrated lipid membranes with CHARMM-GUI (Lee et al. 2016; Wu et al. 2014). Ions were added for charge neutrality. For open conformation GlpG, the POPG lipid membrane simulation system contains 515 lipids for a total of 180.966 atoms; the DOPG system contains 473 lipids, for a total of 178.247 atoms. For closed conformation GlpG, the simulation system contains 514 POPG lipids, for a total of 174.631 atoms.

### Force-Field Description and MD Simulation Protocol

Interactions between atoms of the simulation system were computed with the CHARMM force-field parameters for protein, lipids, and ions (Brooks et al. 2009, 1983; Feller and MacKerell 2000; Klauda et al. 2010; MacKerell et al. 1998, 2004), and with the TIP3P water model (Jorgensen et al. 1983). All MD simulations were performed using NAMD (Kalé et al. 1999; Phillips et al. 2005). Geometry optimization and initial system equilibration were performed using the constraint scheme and velocity rescaling suggested by CHARMM-GUI; briefly, in this scheme, equilibration consists of five steps during which harmonic constraints placed on atoms of the system are gradually released. Following equilibration, all harmonic constraints are switched off and production runs are performed in the *NPT* ensemble (constant number of particles *N*, constant pressure *P* = 1 bar, and constant temperature *T* = 310 K) with a Langevin dynamics scheme (Feller et al. 1995; Martyna et al. 1994). Short-range real-space interactions were treated with a switch function between 10 and 12 Å, and Coulomb interactions were described with smooth particle mesh Ewald summation (Darden et al. 1993; Essmann et al. 1995). Lengths of covalent bonds to H atoms were fixed (Ryckaert et al. 1977).

Heating and first 1 ns of production run were performed with an integration step of 1 fs; for computational efficiency, production runs were performed with a multiple-timestep integration scheme with 1 fs for the bonded forces, 2 fs for short-range non-bonded, and 4 fs for long-range electrostatic interactions (Tuckermann et al. 1992). Coordinates were saved each 1 ps.

### Estimation of the Lipid Bilayer Thickness

The phosphate-to-phosphate (P-P) thickness of the lipid bilayers, *d*_*P-P*_, was estimated as the distance between the peaks of phosphate atoms in the two leaflets. To allow comparison with published reports on the thickness of homogeneous lipid membranes, *d*_*P-P*_ is reported for lipids further than 15 Å from the protein (Bondar 2019; Bondar et al. 2009), and profiles for the distribution of phosphate atoms closer to GlpG are presented separately.

### Sequence Alignment

The GlpG and the *B. subtilis* rhomboid protease sequences were aligned with Clustal Omega (Goujon et al. 2010; Sievers et al. 2011).

### Computations of H-Bond Networks

To identify H-bond networks of GlpG I used Bridge (Siemers et al. 2019), an efficient graph-based algorithm that computes H-bond graphs according to geometric criteria. Two-dimensional H-bond graphs have as nodes groups that H-bond, and, as edges, H-bonds between these groups. H-bonds can be directly between protein groups, directly between protein and lipids, or water-mediated H-bonds (Siemers et al. 2019). Depending on the type of computation tested, Bridge was ~ 68–216 times faster (Siemers et al. 2019) than MDAnalysis (Gowers et al. 2016).

As default, geometric criteria for H-bonding Bridge use a distance of 3.5 Å between the H-bond donor and acceptor heavy atoms, and an H-bond angle of 60° (Siemers et al. 2019). Test computations indicated this combined distance and angle criterion are largely equivalent to a distance of 2.5 Å between the H atom and the acceptor heavy atom (Karathanou et al. 2020).

Here, for the purposes of illustrating how the internal protein H-bond network of open GlpG connects transiently to POPG vs. DOPG lipids, I computed two-dimensional H-bond networks using as H-bond criterion a distance of 3.5 Å between the donor and acceptor heavy atoms. Separately, I used a combined 3.5 Å distance and 60° angle criterion to search for H-bonds of open vs. closed GlpG.

The occupancy of H-bonding reports the percentage of time of the trajectory segment used for analysis during which the criterion used is met.

### Analyses of Protein and Water Dynamics

Computations of Cα root-mean-squared distances (rmsd) were performed with a root-mean-squared fit of each coordinate set relative to the starting crystal structure coordinates of the protein for simulations of open and closed GlpG in POPG; for simulations of open GlpG in DOPG, a coordinate snapshot from an early equilibration step was used as a reference for the rms fit. RMSD, density profiles, and water counts were computed using tcl scripting in VMD.

## Results and Discussion

**Fig. 2:**
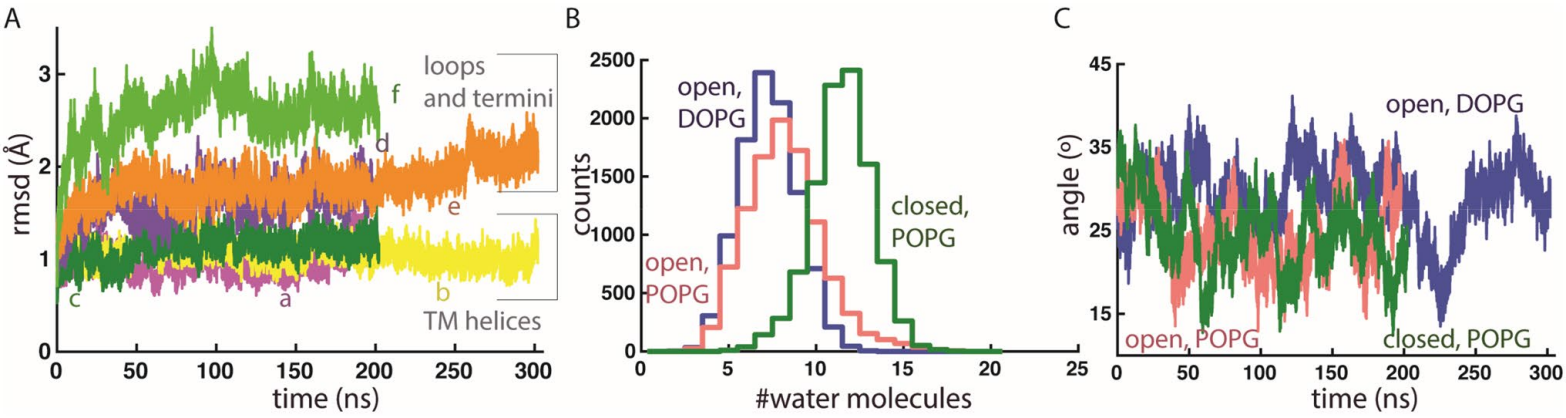
Dynamics of GlpG in POPG and DOPG lipid membranes. **a** Cα rmsd profiles computed for TM helical segments vs. loops and termini for open and closed GlpG. The following letter and color codes are used: open GlpG in POPG, profiles a (purple) and d (violet); open GlpG in DOPG, profiles b (yellow) and e (orange); closed GlpG in POPG, profiles c (green) and f (light green). In all time series, the origin of time indicates the start of the production runs. **b** Histograms of the number of the number of water molecules within 6 Å of S201 for open GlpG in DOPG (blue profile), open GlpG in POPG (light red), and closed POPG in POPG (green profile); for each simulation, histograms were computed based on 10,000 equally spaced coordinate snapshots of the last 100 ns. computed from simulations in POPG (panel **a**) and DOPG (panel **b**). **c** Angle between the principal axis of the protein and the membrane normal computed for open GlpG in DOPG (blue profile) vs. POPG (light red), and for closed GlpG in POPG (green) (Color figure online)

GlpG is structurally stable in all three simulations performed, with Cα root-mean-squared distances of the helical segments within < 2 Å (Fig. 2a). The protein is slightly tilted relative to the membrane normal. In both POPG and DOPG membranes, the tilt of GlpG relative to the membrane normal fluctuates between ~ 15° and ~ 35° (Fig. 2c); a similar fluctuation of the protein tilt was noted in ref. (Zhou et al. 2012). Such fluctuations in the orientation of GlpG relative to the membrane normal indicate orientational dynamics, that is, at least in the absence of a substrate, the precise location of the catalytic site of GlpG along the membrane normal fluctuates in time. Fluctuations in the orientation of GlpG associate with a relatively broad distribution in the number of water molecules that visit the catalytic site of both open and closed GlpG (Fig. 2b).

Fluctuations in orientational dynamics of GlpG could facilitate sampling of an orientation that enables productive interactions with the substrate. However, the orientational dynamics of GlpG might be altered when the substrate approaches.

### The POPG Bilayer Thins Close to Open and Closed GlpG

Recent CHARMM36 simulations of homogeneous bilayers reported for the headgroup-to-headgroup membrane thickness, a value of ~ 38.5 Å for POPG and 43.4 Å for POPE (Shahane et al. 2019). CHARMM36 simulations of a G protein-coupled receptor (GPCR) in homogeneous lipid membranes indicated that, compared to DOPE and DOPC, DOPG lipids have a higher propensity to cluster around the protein (Bruzesse et al. 2018); maps presented for membrane thickness indicate ~ 36 Å for the DOPG membrane far away from the protein (Bruzesse et al. 2018). For comparison, in the same GPCR simulations (Bruzesse et al. 2018), the estimated thickness of the DOPE membrane far away from the protein is ~ 40 Å, and in simulations of a homogeneous DOPE membrane, *d*_*P-P*_ is 40.8 Å (Venable et al. 2015). Taken together, these previous observations on membrane and membrane–protein systems suggest the POPG and DOPG membranes are slightly thinner than the PE counterparts and could engage in close interactions with the protein.

**Fig. 3:**
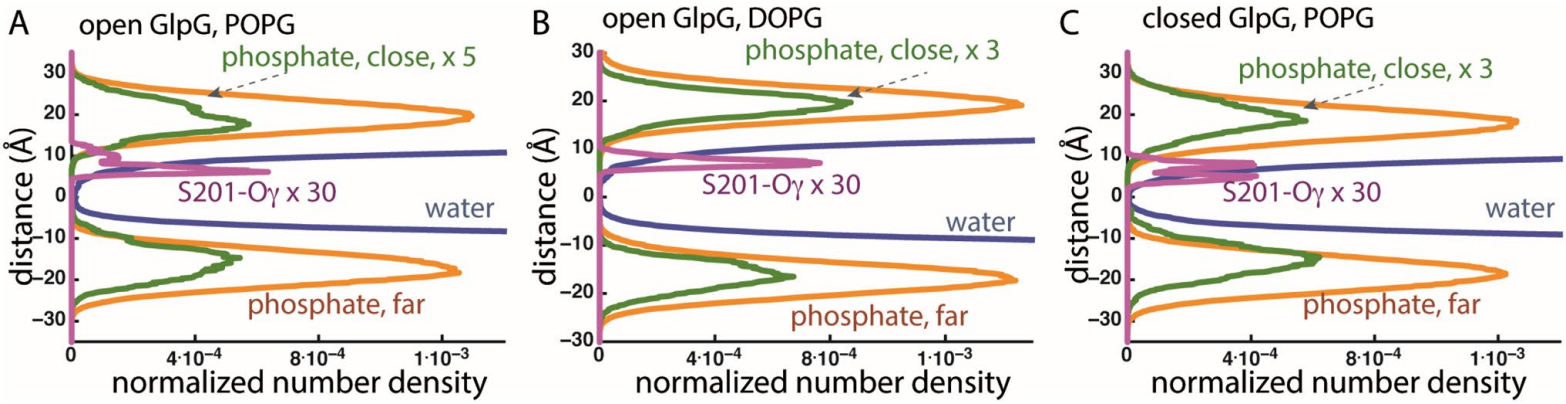
Estimation of the location of the catalytic S201 of GlpG relative to the membrane plane. Normalized number density profiles for lipid phosphate atoms further than 15 Å from the protein are colored brown, lipid phosphate atoms within 15 Å of the protein, green, water oxygen atoms, blue, and for S201-O*γ*, magenta. The distance in Ångstroms gives the coordinate along the membrane normal. Number density profiles were computed from the last 35-36 ns of each simulation and normalized by the volume of the simulation cell. **a**, **b** Number density profiles computed from simulations of open GlpG in POPG membrane (panel **a**) vs. DOPG (panel **b**). **c** Number density profiles computed from simulations of closed GlpG in POPG. Note that in POPG there is nonzero density for lipids at the depth where S201-O*γ* is located, which indicates lipid phosphate atoms can locate, along the membrane normal, at the same depth as S201

Here I find that, far away from GlpG, *d*_*P-P*_ is ~ 37-38 Å for POPG in simulations with open or closed GlpG, and ~ 36.4 Å for DOPG with open GlpG (Fig. 3); these values are compatible with previous computations (Bruzesse et al. 2018; Shahane et al. 2019) and experiments (Pan et al. 2014).

Close to GlpG, H-bonding between lipids and protein groups (see discussion below) associates with local deformations of the membrane (Fig. 1) and normalized number density profiles indicate POPG lipids close to open GlpG sample a membrane depth similar to that of S201 (Fig. 3a). Close to GlpG, *d*_*P-P*_ is ~ 32-33 Å for POPG in simulations with open or closed GlpG (Fig. 3a, c), whereas for DOPG, *d*_*P-P*_ is largely the same close and far away from the membrane (Fig. 3b).

### Lipid Headgroups Visit the Active Site of Open Conformation GlpG and Remain Away from the Active Site of Closed Conformation GlpG

**Fig. 4:**
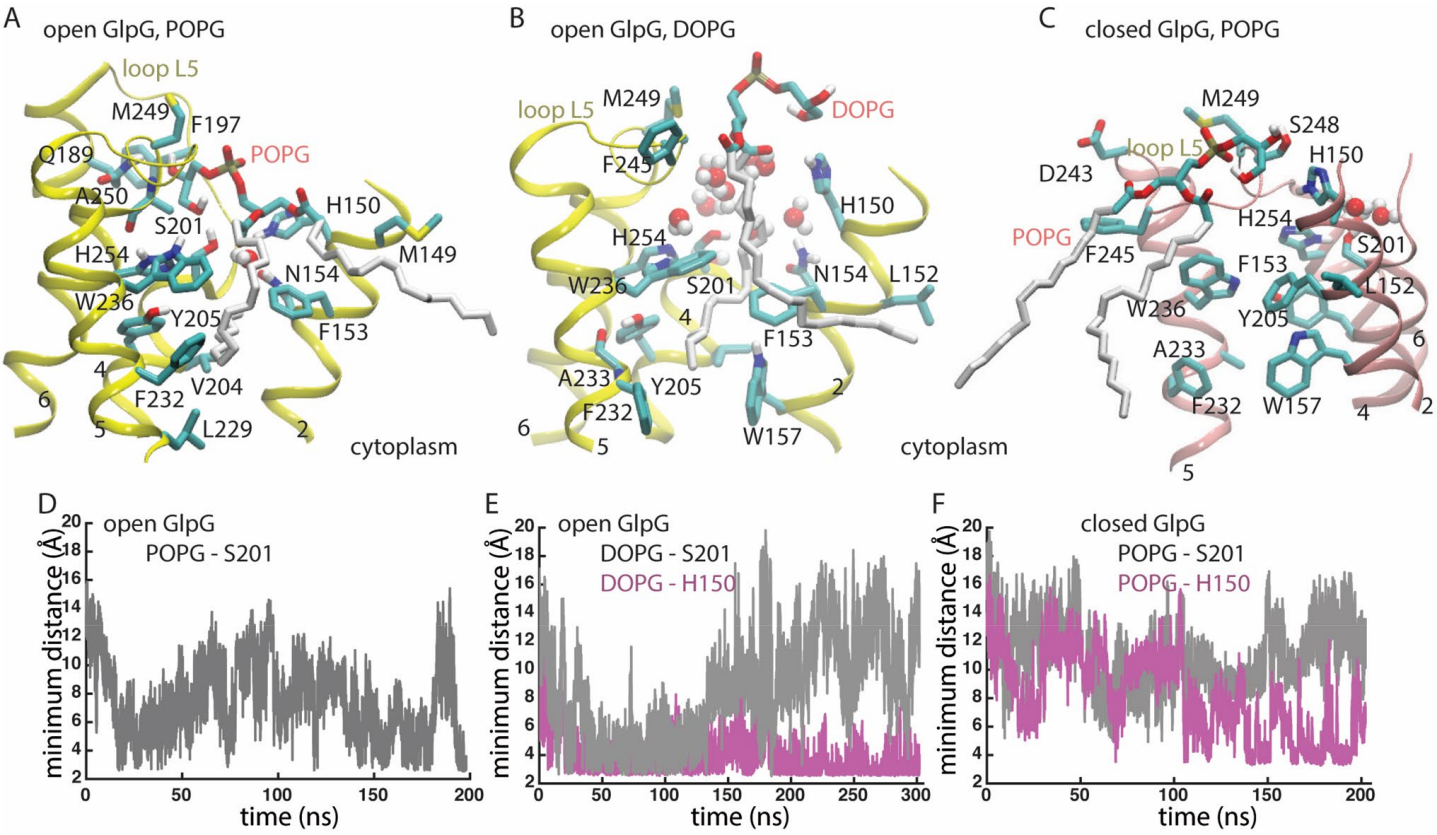
Lipid interactions at the active site of GlpG. **a** Close view of open GlpG in POPG. The hydroxyl group of a POPG lipid interacts directly with the catalytic S201, whereas one of its alkyl chains squeezes in between hydrophobic groups at the interface between TM2 and TM5. A water molecule locates between S201 and N154. **b** Close view of open GlpG in DOPG. H150 interacts with a hydroxyl and a carbonyl group of a DOPG lipid. **c** Molecular graphics of closed GlpG in POPG. **d** Time series of the minimum distance between open GlpG S201-O*γ* and hydroxyl oxygen atoms of POPG. **e** Time series of the minimum distance between open GlpG S201-O*γ* and hydroxyl oxygen atoms of DOPG lipids (light gray), and between H150-N*ε*2 and hydroxyl or carbonyl oxygen atoms of DOPG (magenta). **f** Time series of the minimum distance between closed GlpG S201-O*γ* and hydroxyl oxygen atoms of POPG lipids (light gray), and between H150-N*ε*2 and hydroxyl or carbonyl oxygen atoms of DOPG (magenta)

The crystal structure used for the computations reported here on open conformation GlpG (Ben-Shem et al. 2007) indicates a PG-type lipid bound at the active site, such that its phosphate group is within 2.7 Å of H150 and it makes a bifurcated H-bond with H254 (2.9 Å) and S201 (3.3 Å); an alkyl chain of this lipid packs against F153, F232, and W236. At room temperature in a fluid membrane, both POPG and DOPG lipids can visit the region of the active site of open GlpG (Fig. 4a, b, d, e).

Early during the simulations, a POPG lipid headgroup visits the active site of open GlpG (Fig. 4a, d). The binding is dynamic, and occasionally the distance between the POPG phosphate group and S201-O*γ* is too long for H-bond distance (Fig. 4d); nevertheless, transient encounters between a POPG headgroup and S201 are observed throughout the length of the entire simulation (Fig. 4d). The POPG lipid whose heagroup interacts with S201 docks with one of its alkyl chains between TM helices 2 and 5 (Fig. 4a), the latter of which is thought to function as the gate that controls substrate access to the active site of GlpG (Baker et al. 2007).

H-bond distances between S201-O*γ* and DOPG are observed during the first ~ 130 ns of the simulation (Fig. 4e). For the remaining of the simulation, a DOPG headgroup remains close to the binding site, where it H-bonds to H150 (Fig. 4e). The DOPG lipid whose headgroup interacts with H150 has one alkyl chain docked in the hydrophobic gate of helices 2 and 5 (Fig. 4b).

Taken together, simulations of GlpG in POPG and DOPG membranes suggest that phosphatidylglycerol membranes allow binding of one lipid molecule at the active site of open GlpG, where the lipid engages in H-bonding with S201 or another nearby sidechain, and in hydrophobic packing with sidechains of TM helices 2 and 5 (Fig. 4a, b, d, e).

POPG lipids remain away from the catalytic site of closed GlpG (Fig. 4c, f); nevertheless, during the last ~ 100 ns of the simulation, a POPG headgroup approaches H150 to within H-bond distance (Fig. 4f). This finding is compatible with previous results that POPE lipids tend to remain away from the active site of wild-type closed GlpG (Bondar 2019). Lack of closer lipid interactions could contribute to the active site region of closed GlpG being instead visited by more waters (Fig. 2b).

### A Protein–Lipid H-Bond Network Surrounds open GlpG and Connects Loops L1 and L5 via the Membrane

An unusual feature of GlpG is its long loop L1 that interacts with the membrane (Fig. 1). Loop L1 contains several amino acid residues whose mutation affects enzyme activity, such as the R137A and N154A mutations that abolish and, respectively, strongly reduce proteolytic cleavage of Spitz substrate, and the Y187F mutation that reduces cleavage (Baker et al. 2007). Loop L5 helps control access to the active site (Wang and Ha 2007).

**Fig. 5:**
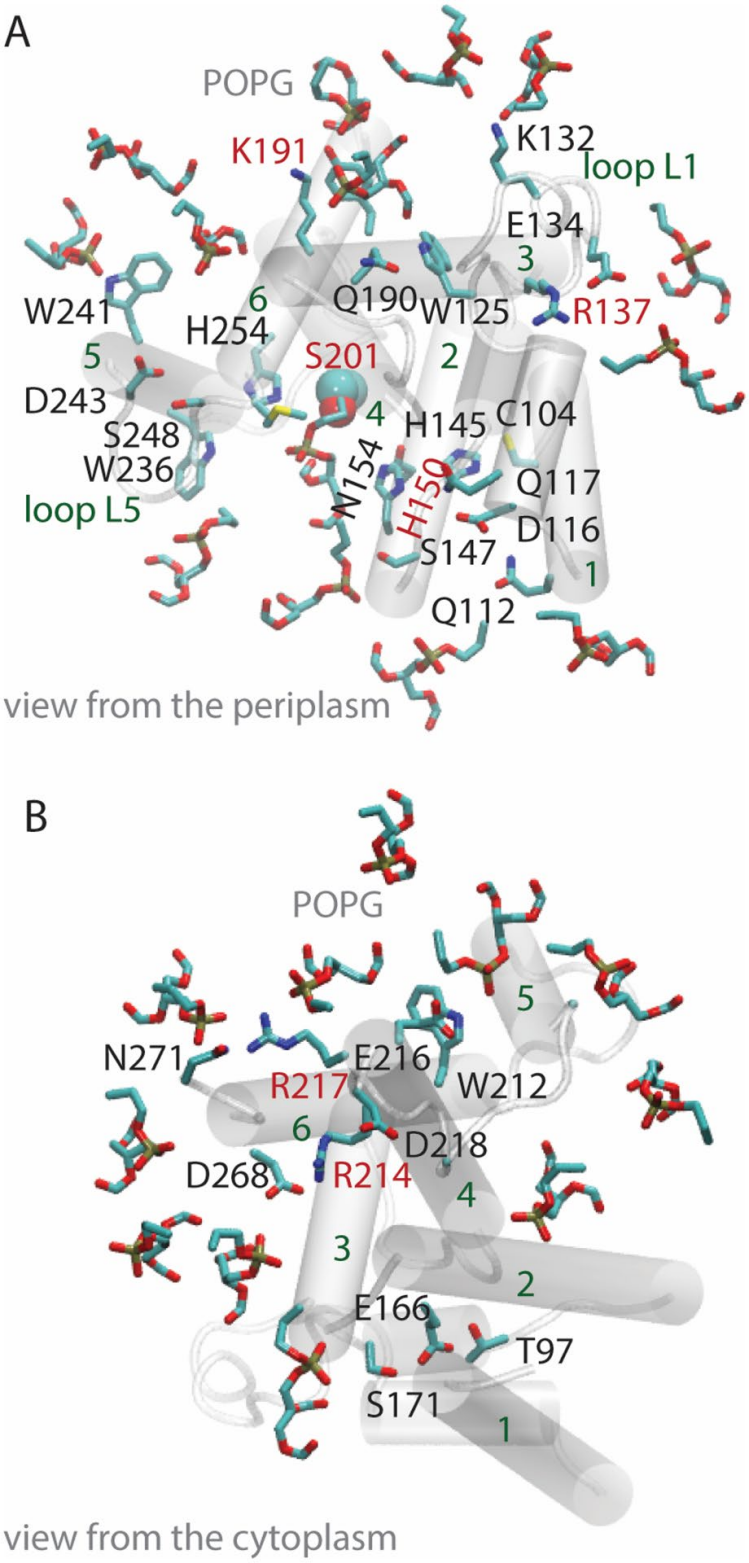
GlpG–lipid interactions in phosphatidylglycerol lipid membranes. Molecular graphics illustrate protein sidechains, and lipid headgroups that are close to the protein. For clarity, H atoms and lipid alkyl chains are not shown. **a**, **b** Open GlpG in POPG lipids, viewed from the periplasmic (panel **a**) vs. the cytoplasmic sides (panel **b**)

**Fig. 6:**
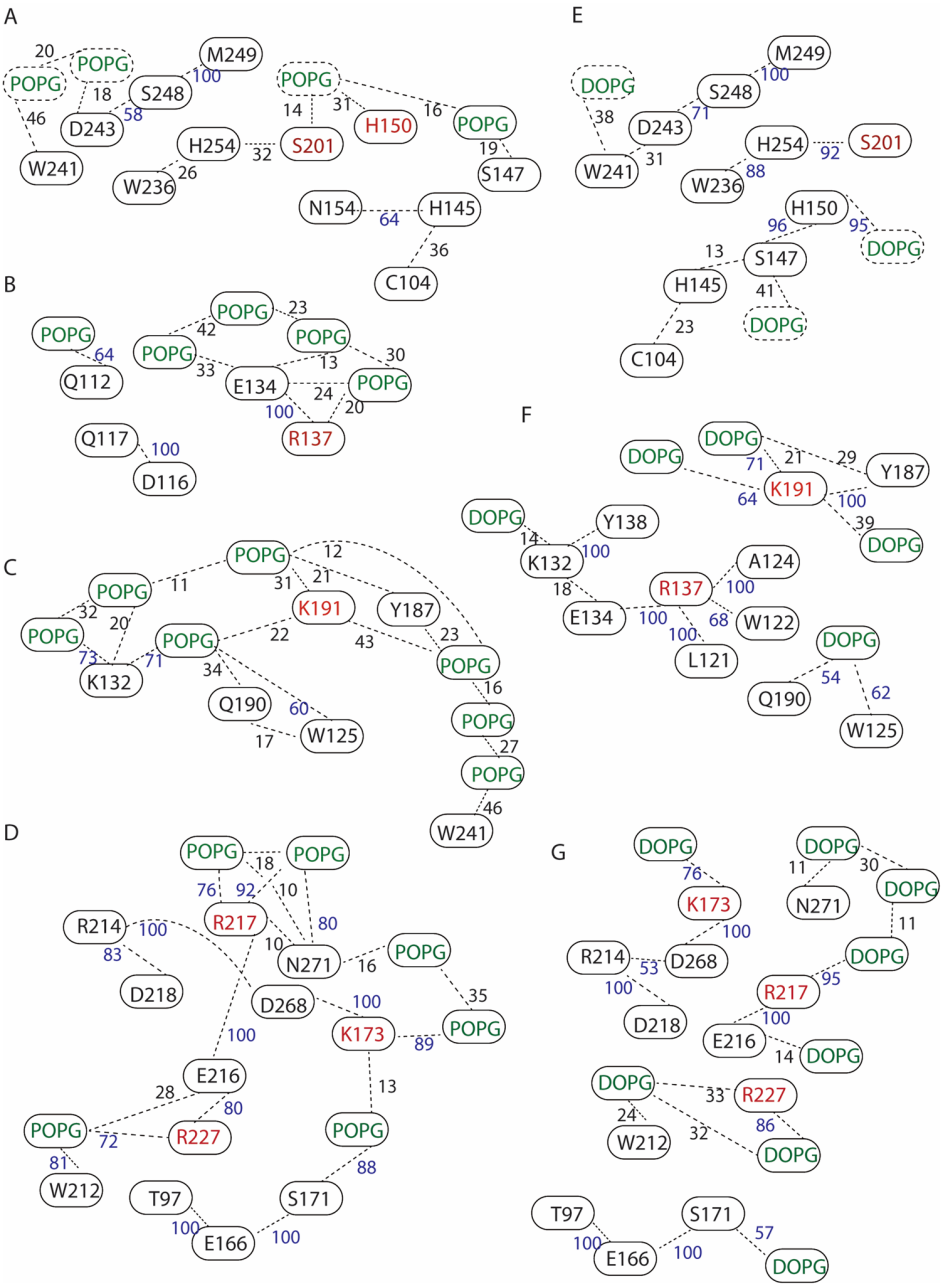
Schematic illustration of selected interactions of open GlpG. Networks of protein groups and lipids with heavy atoms located within distances of 3.5 Å were inspected with VMD (Humphrey et al. 1996) and computed with Bridge (Siemers et al. 2019) using a set of 3500 equally spaced coordinates from the last 35 ns of each simulation. The search included direct H-bonds between protein sidechains, protein sidechains, and protein backbone groups, and between proteins and lipids with phosphate atoms within 6 Å of the protein at the end of each simulation. Numbers indicate the percentage of time of the trajectory segment used for analysis, during which distances are ≤ 3.5 Å; numbers were rounded up to the first integer, and numbers in blue indicate percentages ≥ 50%. **a** Protein–POPG interaction network at the periplasmic side includes the catalytic S201. **b**, **c** Local interaction networks of E134 (panel **b**) and K191 (panel **c**) in POPG. **d** Protein–lipids interaction network at the cytoplasmic side of GlpG in a POPG membrane. K173, R217, and R227, couple the membrane to the internal H-bond network of GlpG. **e** Protein–lipid interaction network at the periplasmic side of GlpG in DOPG. H150 is part of a dynamic H-bond network with protein and lipid headgroups. **f** DOPG–protein interaction networks of K191 and R137. **g** DOPG–protein interaction network at the periplasmic side of GlpG. In both POPG (panel **d**) and DOPG membranes (panel **g**), T97, E166, and S171 are part of a cluster with a lipid molecule

**Fig. 7:**
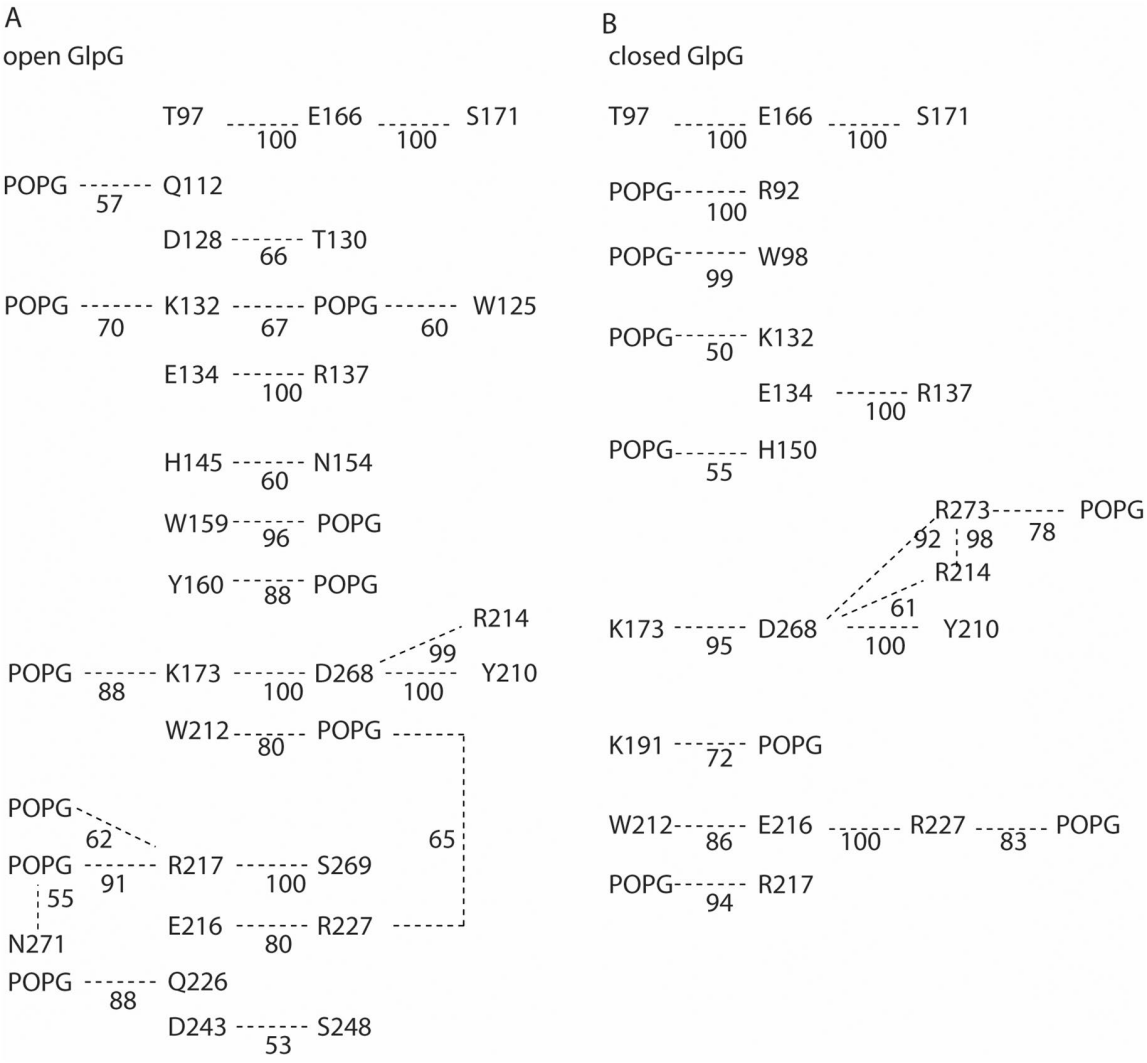
Illustration of selected H-bonds of open vs. closed GlpG in a hydrated POPG bilayer. H-bonds were computed from the last 35 ns and last 36 ns of the open GlpG and closed GlpG simulations, respectively, using as criteria a distance between heavy atoms of ≤ 3.5 Å, and a H-bond angle of ≤ 60°. For simplicity, only lipids within 6 Å of the protein at the end of the simulation were included in the search for H-bonds, and only those H-bonds are shown that are present for at least 50% of the trajectory segment used for analyses. **a**, **b** Schematic illustration of selected H-bond networks of open (panel **a**) vs. closed GlpG (panel **b**)

In both POPG and DOPG membranes, protein groups and lipids participate in extensive networks of interactions whereby H-bonds may form transiently, such that protein segments interconnect via the membrane (Figs. 5, 6, 7).

At the periplasmic side, S201 is part of a dynamic H-bond network that extends to POPG lipids (Fig. 6a); in DOPG, S201 is part of a local H-bond cluster without direct connections to lipids, but two groups from the vicinity of the catalytic site, H150 and D243, are part of dynamic H-bond networks that connect to lipids (Fig. 6e).

K191 (Fig. 5a) is part of a dynamic protein-POPG network that includes W241 of the cap loop L5 (Fig. 6c), and, in DOPG, it interacts with three lipid headgroups and with Y187 (Fig. 6f). The involvement of K191 in H-bond networks with POPG and DOPG lipid headgroups is compatible with previous work indicating K191 H-bonds with lipids in simulations with GlpG embedded in DMPC, DOPC, POPC, POPE, and POPE:POPG membranes (Bondar 2019).

At the cytoplasmic side, R217, R227, and K173, anchor GlpG in the membrane and connect the membrane to an internal H-bond network (Figs. 5b, 6d, g).

Within the trajectory segments used for analyses of the interaction networks, only some of the protein groups remain within H-bond distance all the time—the most prominent example being the triad T97—E166—S171 (Fig. 6d). For many of the interactions, H-bond distances are sampled some of the time.

Values of the H-bond occupancies, and details of the local arrangements of protein groups and lipids, might change with time, length of the simulations, or criteria used to define H-bonding. The qualitative picture will, however, likely remain largely the same: Tips of helical segments, and loops L1 and L5, are part of dynamic networks whereby protein groups and lipid headgroups come transiently to within distances that allow H-bonding. Lys and Arg groups anchor GlpG in the membrane and couple internal protein H-bond networks to the membrane.

### High-Occupancy H-Bond Networks of Open vs. Closed GlpG in POPG

To compare H-bond networks of open and closed GlpG, the schematic diagram presented in Fig. 7 illustrates high-occupancy H-bonds. These protein–protein and protein–lipid H-bonds were identified with a commonly used criterion of 3.5 Å for the distance between heavy atoms, and a H-bond angle of 60°; only H-bonds present during at least 50% of the trajectory segments used for analyses are shown.

With this more stringent computation of the H-bond network, K132, R217, and R227 remain sites where POPG lipids H-bonds to the both open and closed GlpG (Fig. 7). In open GlpG, K191 is within H-bond distance of three lipid headgroups all of which have occupancies below 50% (Fig. 6); in closed GlpG, K191 has a high-occupancy lipid H-bond (Fig. 7b).

Overall, the qualitative picture remains largely similar to that discussed above for open GlpG in POPG vs. DOPG membranes (Fig. 6): The internal H-bond network of GlpG anchors to lipids at multiple sites.

## Conclusions

I reported on atomistic simulations of open and closed conformations of the *E. coli* GlpG rhomboid protease embedded in POPG and DOPG membranes. These membrane models were chosen to dissect mechanisms by which phosophatidylglycerol lipids, which are a major component of bacterial membranes, may impact catalytic activities of bacterial rhomboids.

POPG and DOPG lipid membranes are relatively thin, their estimated thickness of ~ 37-38 Å being relatively close to that of the cytosolic *E. coli* membrane. Both POPG and DOPG lipid headgroups H-bond transiently with protein groups, including, in the case of open GlpG, with the catalytic S201, which is located relatively deep in the membrane plane (Figs. 1, 3, 4a–c).

H-bonding of K191 was found to contribute to membrane thinning: in simulations of open GlpG in POPE, average membrane thinning was smaller by almost 2 Å in K191A than in the wild-type protein (Bondar 2019). Here, H-bonding between K191 and lipids is sampled in all simulations (Figs. 6c, f, 7). Likewise, R217 and R227 anchored GlpG to lipids (Figs. 6d, g, 7), as observed in previous simulations regardless of the conformation of GlpG and of the lipid membrane composition (Bondar 2019).

By contrast to these membrane-exposed Lys and Arg sidechains, lipid interactions of groups at the active site of GlpG depend on the protein conformation: POPG and DOPG could approach the active site of open GlpG (Fig. 4a, b, d, e), but POPG remained away from the active site of closed GlpG (Fig. 4c, f).

The finding here that POPG and DOPG may visit the active site of open GlpG raises the question as to why, when motions of GlpG were studied previously in a mixed POPE:POPG lipid membrane, lipids remained outside of the region of the catalytic groups, and transient binding of POPG at the active site was not observed (Bondar 2019)—instead, POPE lipids interacted with the cap loop L5, which could prevent POPG lipids from approaching the active site (Bondar 2019). It thus appears that interactions between PG lipids and the active site of GlpG depends on details of the lipid membrane composition. That different lipid species found in a mixed lipid bilayer can have preferred binding sites on a protein surface was observed before in simulations of a potassium ion channel (Duncan et al. 2020).

**Fig. 8:**
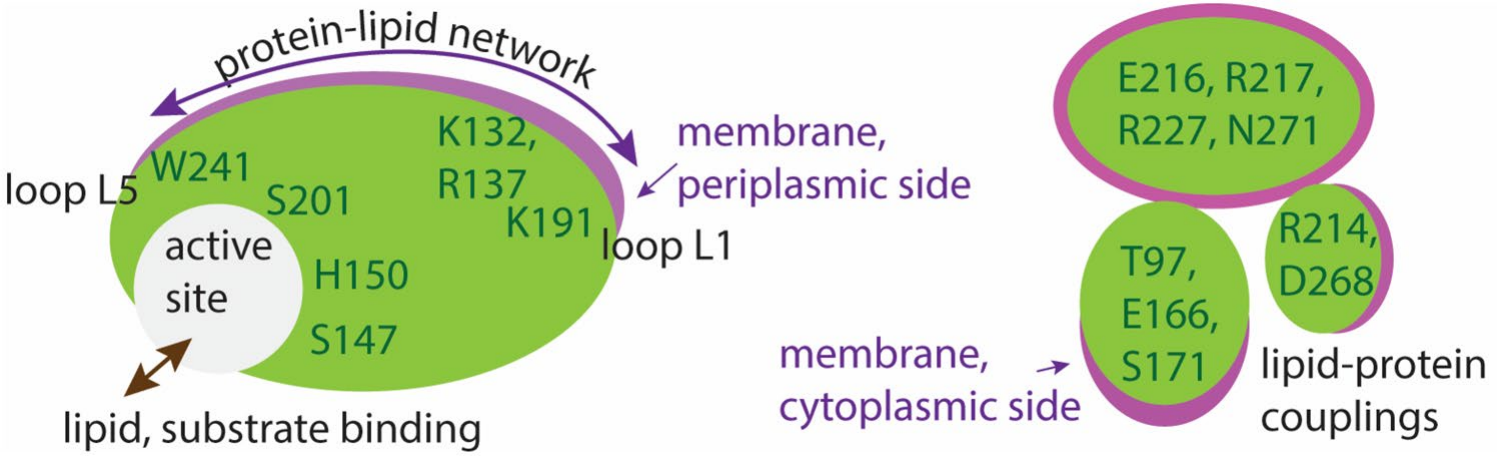
Schematic representation of a potential mechanism by which lipids may impact the reaction coordinate of GlpG. Green and magenta shading indicate protein and membrane, respectively. Selected amino acid residues included in the schemes are part of H-bond networks illustrated in Fig. 7. At the periplasmic side, protein groups from the cap loop L5 and from loop L1 are part of dynamic H-bond clusters that may extend through the membrane and bridge the two regions of the protein. Stable lipid binding at the active site could compete with substrate binding. At the periplasmic side, internal protein H-bond clusters couple to the lipid membrane via dynamic H-bonding

Lipid headgroups and protein groups interact mostly dynamically, in networks that typically include multiple potential H-bond partners (Figs. 5–8). At the periplasmic side of open GlpG, groups of loops L1 and L5 can be part of distinct local protein–lipid networks (Fig. 6b, f), or can participate in a common dynamic network that extends from K191 to K132 and W241 (Fig. 6c). At the cytoplasmic side, protein H-bonding couples to the membrane at sites T97-E166-S171, K173-D168, R217, and R227 (Fig. 6d, g). Such local protein–lipid interaction networks likely contribute to the impact of the lipid membrane composition on the catalytic activity of GlpG (Fig. 8) and might be a more general feature of intramembrane proteases. Indeed, earlier simulations of a presenilin model indicated direct H-bonding between the two catalytic aspartic groups and a POPE lipid headgroup (Kong et al. 2015), and negatively charged lipids are known to bind to proteins (Pyöry and Vattulainen 2016).

The H-bond dynamics of carboxylate and histidine groups depend on their protonation states, and changes in protonation states can impact protein and internal water dynamics (Bondar and Smith 2017; del Val et al. 2014). Protonation states of rhomboid proteases are poorly described. As noted before, inspection of static GlpG crystal structures would suggest H254 singly protonated on Hδ (Bondar et al. 2009; Uritsky et al. 2012), as considered here. Another site of potential interest for proton binding is E166, whose carboxylate group is part of an interhelical H-bond cluster with T97 and S171 (Figs. 6d, g, 7). Such interhelical carboxylate-hydroxyl H-bonds are often common at proton-binding sites of membrane transporters and receptors (Bondar and Lemieux 2019; Bondar and Smith 2017; del Val et al. 2014) but, whether could E166 binds a proton, remains unclear.

Water enters deep in the membrane, such that water molecules interact with protein groups at the active site of GlpG (Figs. 2b, 3, 4a, b). Exposure to water at a site deep in the membrane, fluctuations in the protein tilt (Figs. 2c), and overall dynamic networks of protein–lipid H-bonds suggest the membrane environment of GlpG is dynamic. The orientational dynamics of GlpG in the membrane might participate in facilitating productive interactions with the substrate, e.g., by enabling GlpG to reorient rapidly when the substrate approaches the active site, such that a stable enzyme–substrate might be established.

How might PG lipids interfere with GlpG cleavage of substrate? The atomistic simulations presented here and elsewhere (Bondar 2019) suggest lipids could impact the reaction coordinate of GlpG via a ligand-like behavior, whereby they bind at the active site of GlpG potentially competing with docking of a transmembrane substrate (Fig. 8), they anchor the protein in the membrane by binding at sites such as K191, and bridge remote membrane-exposed protein groups of loops L1 and L5—which could provide long-distance conformational couplings. An important role of lipid binding for the conformational dynamics of GlpG is supported by the recent observation that membrane-exposed groups are part of a conserved group of amino acid residues important for substrate interactions (Mihaljević and Urban 2020).

Differences in protein sequence would impact protein–lipid interactions, such that bacterial rhomboids might respond differently to a particular lipid environment. For example, in the *B. subtilis* rhomboid, which is active in PG lipids (Urban and Wolfe 2005), both K191 of loop L1 and D243 of the cap loop L5 are replaced by Gly groups. Since the K191A mutation associates with reduced membrane thinning, and in D243A, the cap loop L5 may sample closer interactions with the protein (Bondar 2019), differences in the local amino acid sequences of *E. coli* and *B. subtilis* rhomboids could lead to different lipid–protein interactions of the two rhomboids, including different orientational dynamics of the protease in the membrane, and different dynamics at the active site. To conclude on the mechanism by which lipids shape reaction coordinates of rhomboid proteases, free energy computations of electronic structure changes during proteolytic bond cleavage various membranes would be necessary.

A caveat affecting study of the reaction coordinate of GlpG is that its physiological substrate remains unknown, though recent work suggests GlpG might be involved in gut colonization by *E. coli* (Russell et al. 2017). In the future, advances in computational approaches, which allow simulations with complex protein–lipid mixtures (Corradi et al. 2018; Enkavi et al. 2019; Marrink et al. 2019), might make it feasible to derive a molecular movie of dynamic encounters between rhomboid proteases and substrates in realistic, crowded membrane models. As proteins are thought to have unique preferences for the composition of the first shell of lipids (Corradi et al. 2018), simulations might provide clues as to why rhomboids from various bacteria respond differently to changes in the lipid composition (Urban and Wolfe 2005). Knowledge of the physiological substrate of GlpG will enable experiments and computations to dissect the role of specific lipids in shaping the reaction coordinate of proteolytic substrate cleavage by rhomboid proteases.
